# Expression of Concern: Long non-coding RNA ROR1-AS1 incudes tumorigenesis of colorectal cancer by affecting Wnt/β-catenin signaling pathway

**DOI:** 10.1042/BSR-20191453_EOC

**Published:** 2020-07-07

**Keywords:** Long noncoding RNA, ROR1-AS1, colorectal cancer, Wnt/β-catenin signaling pathway

The Editorial Office has been made aware of potential issues surrounding the scientific validity of this paper, hence has issued an expression of concern to notify readers whilst the Editorial Office investigates.

